# Demethylation restores SN38 sensitivity in cells with acquired resistance to SN38 derived from human cervical squamous cancer cells

**DOI:** 10.3892/or.2012.1628

**Published:** 2012-01-11

**Authors:** TETSUJI TANAKA, TAO BAI, SAORI TOUJIMA, TOMOKO UTSUNOMIYA, TOSHIHIDE MATSUOKA, AYA KOBAYASHI, MADOKA YAMAMOTO, NORIYUKI SASAKI, YUKO TANIZAKI, HIROTOSHI UTSUNOMIYA, JUNKO TANAKA, KAZUNORI YUKAWA

**Affiliations:** 1 Santamaria Hospital, Ibaraki, Osaka 567-0884, Japan; 2 Department of Neurology, The University of Chicago, Chicago, IL 60637, USA; 3 Department of Obstetrics and Gynecology, Wakayama Medical University, Wakayama 641-0012; 4 Department of Pathology, Wakayama Medical University, Wakayama 641-0012; 5 Graduate School of Intercultural Studies, Kobe University, Nada-ku, Kobe 657-8501; 6 Department of Physiology, Faculty of Pharmacy, Meijo University, Tempaku-ku, Nagoya 468-8503, Japan

**Keywords:** SN38, CPT-11, drug-resistance, methylation, death-associated protein kinase, squamous cell carcinoma, cervical carcinoma

## Abstract

Using seven monoclonal SN38-resistant subclones established from ME180 human cervical squamous cell carcinoma cells, we examined the demethylation effects of 5-aza-2′-deoxycytidine (5-aza-CdR) on the SN38-sensitivity of the cells as well as the expression of death-associated protein kinase (DAPK) in the SN38-resistant cells. The DAPK expression levels were evaluated among parent ME180 cells, SN38-resistant ME180 cells and cisplatin-resistant ME180 cells by methylation-specific DAPK-PCR, quantitative RT-PCR and western blot analysis. The SN38-resistant cells co-treated with SN38 and 5-aza-CdR strongly exhibited enhanced SN38-sensitivities resembling those found in the parent cells. In the SN38-resistant subclones, no relationships were found between the restored SN38 sensitivity and hypermethylation of the DAPK promoter, DAPK mRNA expression, DAPK protein expression and induction of DAPK protein after 5-aza-CdR treatment, unlike the strong suppression of 5-aza-CdR-induced DAPK protein expression in the cisplatin-resistant subclones. These findings indicate that reversibly methylated molecules, but not DAPK, may regulate SN38 resistance, and that demethylating agents can be strong sensitizing anticancer chemotherapeutic drugs for SN38-resistant cancers.

## Introduction

Recent progress in anticancer chemotherapy for advanced cancer patients has improved the long-term overall survival rates. Consequently, acquired drug-resistance during chemotherapy is now the largest clinical problem that inhibits complete remission in advanced cancer patients. The prognosis of patients with recurrent cancers or advanced malignant tumors will be remarkably improved if the mechanisms involved in the acquisition of anticancer drug-resistance are clarified and therapies to overcome drug-resistance or to restore drug-sensitivity are established.

Irinotecan HCl (CPT-11), an anticancer prodrug, is converted into its main active metabolite, SN38, by carboxyl esterase in the body. SN38 is the most powerful inhibitor of topoisomerase I and shows strong antitumor effects by antagonizing DNA synthesis (1). CPT-11 has been used clinically in various cancer chemotherapies for uterine (2–4), ovarian (5,6), lung (7), colorectal (8,9) and gastric cancers (10) and malignant lymphoma (11), and high response rates of these therapies have been reported. In our clinical experience, combined chemotherapies with CPT-11 showed a 72.4% response rate for gynecologic malignancies (12) and neo-adjuvant chemotherapy with CPT-11 for advanced cervical squamous cancer patients showed a 100% response rate (4), suggesting that CPT-11 must be one of the most clinically effective anticancer drugs. Although many studies on the mechanisms of anticancer drug-resistance have been reported, few investigations have been carried out on the mechanisms underlying CPT-11-resistance or SN38-resistance.

Death-associated protein kinase (DAPK) cDNA was isolated from human cervical carcinoma cells as a positive mediator of apoptosis triggered by IFN-γ (13). Recent investigations have revealed that DAPK functions as a Ca^2+^/calmodulin-dependent serine/threonine kinase to regulate cell death or survival (13–28). However, the physiological functions of DAPK have not been fully clarified. Loss of DAPK expression has been implicated in tumorigenesis and metastasis (15,16,25), suggesting a crucial role for DAPK in the apoptotic process under pathological conditions. On the other hand, inhibition of DAPK expression in HeLa cells, 3T3 fibroblasts, primary human vascular smooth muscle cells and various human uterine cancer cells using antisense DAPK or small-interfering RNAs (siRNAs) for DAPK was found to increase apoptosis (18,24,27,28).

In the human cervical squamous cell carcinoma cell line ME180, which exhibits hypermethylation of normally unmethylated CpG islands in the promoter region of the DAPK gene (25), DAPK protein expression is constitutively suppressed but can be strongly induced by treatment with the demethylating agent 5-aza-2′-deoxycytidine (5-aza-CdR) (29). However, in ME180-derived cisplatin (CDDP)-resistant cell lines, DAPK protein expression cannot be induced by treatment with 5-aza-CdR. Since DAPK mRNA is expressed in the CDDP-resistant cells in a similar manner to the ME180 parent cells, the strong suppression of DAPK protein induction after 5-aza-CdR treatment may be a result or an indicator of acquired CDDP-resistance. In the human endometrial adenocarcinoma cell line HHUA, in which DAPK protein is highly expressed (25), targeted knockdown of DAPK protein expression with DAPK siRNAs enhanced 5-fluorouracil-sensitivity but not etoposide-sensitivity, suggesting that DAPK protein expression levels can regulate cellular sensitivity to some anticancer drugs (30).

Previously, we established seven monoclonal SN38-resistant subclones from ME180 cells (31) to investigate the molecular mechanisms underlying acquired SN38-resistance in cervical cancer and squamous cell carcinoma cells, and reported that all the SN38-resistant cells showed strong resistance against radiation-induced cell death (32). In the present study, we examined the effects of 5-aza-CdR treatment on the DAPK expression and SN38-sensitivity in these SN38-resistant subclones to investigate the potential therapeutic applications of regulating the DAPK expression and SN38-sensitivity of SN38-resistant cancers.

## Materials and methods

### Cell lines and culture

The human cervical squamous cell carcinoma cell line ME180 was purchased from the JCRB Cell Bank (Japan Collection of Research Bioresources Cell Bank, Tokyo, Japan). All cells were cultured in Opti-MEM (Invitrogen Corp., Carlsbad, CA, USA) supplemented with 5% fetal bovine serum (Equitech Bio Inc., Ingram, TX, USA), 100 U/ml penicillin, 100 μg/ml streptomycin and 0.25 μg/ml fungizone (Invitrogen) in 5% CO_2_/95% air at 37˚C.

### Establishment of SN38-resistant subclones from ME180 cells

SN38, the most active metabolite of CPT-11, was a gift from Yakult Honsha Co., Ltd. (Tokyo, Japan). To establish SN38-resistant subclones, ME180 cells were cultured with various concentrations of SN38 for 3–5 weeks, and the surviving cells were collected. This collection procedure after SN38 exposure was repeated four times. Finally, seven single cell-derived SN38-resistant subclones, designated SN38r1, SN38r2, SN38r7, SN38r8, SN38r9, SN38r10 and SN38r12 were established by the limiting dilution method. The monoclonality of each SN38-resistant subclone was confirmed by chromosome analysis (31). The establishment of these SN38-resistant subclones took 1 year.

### SN38 sensitivity assay

Cell viability was assayed using a Cell Counting kit (Dojindo Chemical Laboratory Co., Ltd., Tokyo, Japan) according to the manufacturer's instructions. The inhibitory effects of SN38 on cell growth were assayed as follows. Cells in the log phase were detached with 0.25% trypsin/1 mM EDTA (Invitrogen), and cultured overnight in 96-well plates (5,000 cells/well). On Day 2, various concentrations of SN38 were added to the cells. On Day 4, cell viability was evaluated using the kit and expressed as the percentages of viable cells (%) relative to the mean number of viable unstimulated cells. All experiments were performed three times to verify the results. The data are presented as mean ± SD, and comparative data (n=6) were analyzed by ANOVA.

### Effects of the methyltransferase inhibitor 5-aza-CdR on the SN38-sensitivities of the parent cells and SN38-resistant subclones

Approximately 5,000 ME180 parent cells or ME180-derived SN38-resistant subclones were seeded in 0.1 ml of Opti-MEM in 96-well culture plates and incubated for 24 h. The medium was then replaced with medium containing the designated concentrations of SN38 with or without 1 μM 5-aza-CdR (Sigma, St. Louis, MO, USA) and the cells were incubated for a further 48 h. Next, the viable cell numbers were determined using the Cell Counting kit. The absorbance was measured at 450 nm using a 96-well plate reader (Dainihon-Seiyaku Co., Osaka, Japan). The absorbance obtained from the control cells without drug administration was set as 100% viability. All experiments were performed three times to verify the results. The data are shown as mean ± SD, and comparative data (n=6) were analyzed by ANOVA.

### RT-PCR and quantitative real-time RT-PCR of DAPK and DNA methyltransferase (DNMT) genes

Total RNA was isolated from cultured cells using the TRIzol reagent (Invitrogen) for RT-PCR analyses or an RNeasy Mini Kit (Qiagen Inc., Valencia, CA, USA) for real-time RT-PCR analyses. Aliquots containing 1 μg of total RNA were pretreated with DNase I (Invitrogen) and then subjected to cDNA synthesis using a reverse transcriptase kit (Bio-Rad Laboratories, Hercules, CA, USA) in a reaction volume of 20 μl. Each cDNA product was diluted to 100 μl. The PCR reaction mixture (25 μl) contained 5 μl of diluted cDNA, 0.125 μl of Hotstart polymerase (Qiagen Inc.), 0.2 mM dNTP, 1xQ solution and 0.5 μM primers. The primers used for DAPK, DNMT1, DNMT3A and DNMT3B have been described in a previous report (29). β-actin was evaluated as a positive control for the mRNA amount. For RT-PCR analyses, an initial hot start at 95˚C for 15 min was followed by 35 amplification cycles of 30 sec at 94˚C, 30 sec at the annealing temperature and 60 sec at 72˚C. The annealing temperatures were 55˚C for DAPK and 60˚C for DNMT1, DNMT3A and DNMT3B. The PCR products were electrophoresed on 1.5–2.0% agarose gels at 100 V for ~30–40 min and visualized by staining with 5 μg/ml ethidium bromide. Quantitative real-time RT-PCR analyses were performed using an iCycler (Bio-Rad Laboratories). The PCR reaction mixture (25 μl) contained 2.5 μl of diluted cDNA, 12.5 μl of iQT™ SYBR-Green Supermix (Bio-Rad Laboratories) and 0.5 μM of the above-described primers. An initial hot start at 95˚C for 3 min was followed by 40 cycles of amplification at 95˚C for 30 sec, 55˚C for 30 sec and 72˚C for 60 sec. The relative values of DAPK mRNA in the ME180 parent cells and SN38-resistant subclones were calculated based on the 2^−ΔΔCT^ method, where ΔΔC_T_ was calculated from the following formula:

$$\begin{array}{c} \Delta \Delta {\text{C}}_{\text{T}}=\Delta {\text{C}}_{\text{T SN}38-\text{resistant subclones}}-\Delta {\text{C}}_{\text{T ME}180\;\text{parent cells}}=\\ {\left({\text{C}}_{\text{T DAPK}}-{\text{C}}_{\text{T actin}}\right)}_{\text{SN}38-\text{resistant subclones}}-{\left({\text{C}}_{\text{T DAPK}}-{\text{C}}_{\text{T actin}}\right)}_{\text{ME}180\;\text{parent cells}} \end{array}$$

### Western blot analysis of DAPK protein expression

The ME180 parent cells and SN38-resistant subclones were incubated with 1–10 μM 5-aza-CdR for 48 or 96 h, after which they were harvested and lysed with 0.3 ml of lysis buffer (Sigma). The protein contents of the cell lysates were quantified using a Coomassie Plus Protein assay (Pierce Biotechnology Inc., Rockford, IL, USA) and aliquots (25 μg total protein) were dissolved in Laemmli SDS-PAGE sample buffer prior to separation by 7.5% SDS-PAGE. The separated proteins were transferred to a polyvinylidene fluoride membrane (ATTO Corp., Tokyo, Japan) using a wet transfer method. The membrane was blocked with 5% skim milk for 1 h at room temperature and subsequently incubated with a mouse monoclonal anti-human DAPK antibody (clone 55; 1:5000 dilution; Sigma) for 1 h at room temperature. After washing with TBS-T (20 mM Tris-HCl pH 7.6, 0.3 M NaCl, 0.1% Tween-20), the membrane was incubated with a horseradish peroxidase-conjugated rabbit anti-mouse secondary antibody (Sigma) for 1 h at room temperature. The bound antibodies were detected with an ECL Plus kit (Amersham Pharmacia Biotech, Uppsala, Sweden) and the membrane was scanned using a chemiluminescence imaging system (Luminocapture AE6955; ATTO Corp.).

### DAPK-methylation-specific PCR (DAPK-MS-PCR)

Genomic DNA was isolated from cultured cells using a SepaGene kit (Sanko-Junyaku Ltd., Tokyo, Japan) according to the manufacturer's instructions. The DNA concentrations were calculated from the UV absorptions at 260 and 280 nm. DAPK-MS-PCR was performed as previously described (29). Briefly, a bisulfite-modified DNA was used as a template for stage I PCR amplification to generate a 209-bp fragment of the DAPK gene that included a portion of its CpG-rich promoter region. The stage I PCR primers used recognized the modified DNA but could not discriminate between methylated and unmethylated alleles. The stage I PCR amplification was carried out as follows: 95˚C for 15 min; 35 cycles of denaturation at 94˚C for 1 min, annealing at 58˚C for 150 sec and extension at 72˚C for 150 sec; and a final extension at 72˚C for 10 min. The stage I PCR products were diluted 1:50, and 5 μl was subjected to stage II PCR amplification using primers specifically designed for methylated or unmethylated DNA in the promoter region of the DAPK gene. The primers used for the stage I and II PCR amplifications were previously described (29). For stage II PCR, the annealing temperature was increased to 65˚C and the annealing time was reduced to 90 sec for 40 cycles. The stage II PCR amplified 153-bp and 106-bp products from the methylated and unmethylated DAPK genes, respectively. Finally, the PCR products were electrophoresed on a 2.0% agarose gel at 100 V for ~30–40 min, and visualized by staining with 5 μg/ml ethidium bromide.

## Results

### Effects of demethylation on the SN38-sensitivities of the SN38-resistant subclones

**Figure 1:**
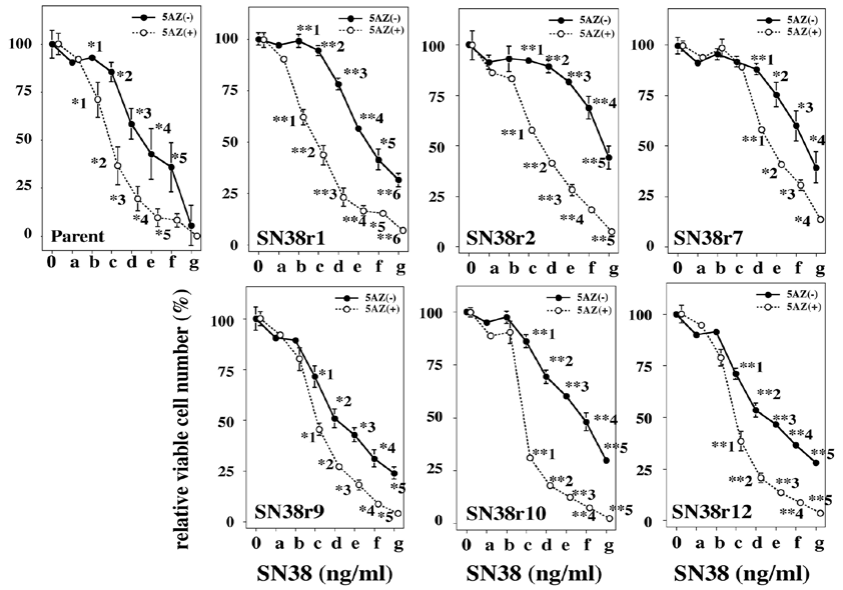
Effects of 5-aza-CdR treatment on the SN38-sensitivities of the parent cells and six SN38-resistant subclones. The solid lines with closed circles are the SN38-sensitivity curves of cells treated with SN38 alone and the dotted lines with open circles are the SN38-sensitivity curves of cells concurrently treated with SN38 and 5-aza-CdR. All the cells treated with 5-aza-CdR and SN38 show significantly higher SN38-sensitivities than the cells treated with SN38 alone. The final concentrations (ng/ml) of SN38 indicated as 0 and a-g at the bottom of the figures are 0, 0.256, 1.28, 6.4, 32, 160, 800 and 4000 ng/ml, respectively. ^*^p<0.05, ^**^p<0.01.

To examine whether the acquired SN38-resistance of the SN38-resistant subclones was caused by aberrant methylation of gene promoters, we analyzed the effects of demethylation on the SN38-sensitivities of six SN38-resistant subclones (Fig. 1). Preliminary culture experiments with 5-aza-CdR (0–20 mM) treatment showed no growth-inhibitory effects on the ME180 parent cells (data not shown). Next, we treated the parent cells and SN38-resistant subclones with various concentrations of SN38 and 10 μM 5-aza-CdR for 48 h to examine the effects of concurrent treatment with 5-aza-CdR and SN38 on the SN38-sensitivities. As shown in Fig. 1, the parent cells co-treated with SN38 and 5-aza-CdR showed significantly increased SN38-sensitivity compared with the parent cells treated with SN38 alone. All six SN38-resistant subclones examined showed 25–125-fold higher SN38-sensitivities when co-treated with SN38 and 5-aza-CdR compared with the same subclones treated with SN38 alone.

### Transcription of the DAPK gene and expression of DAPK protein in the parent cells and SN38-resistant subclones

**Figure 2:**
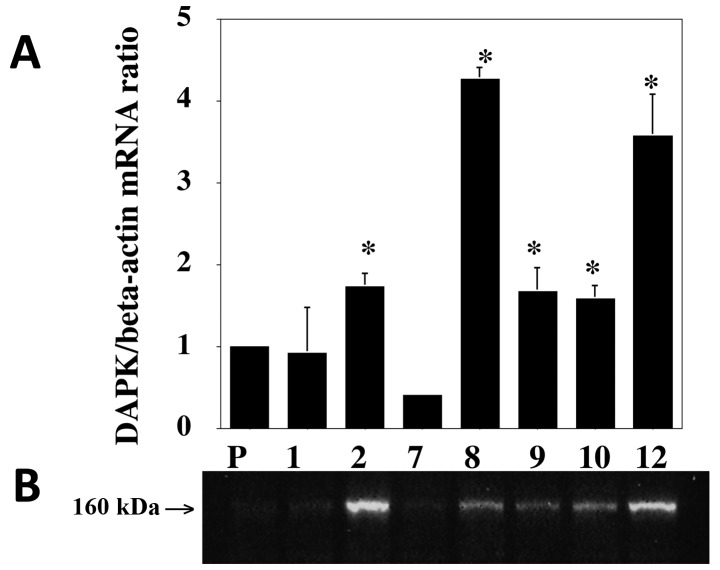
Quantitative real-time RT-PCR and western blot analyses of DAPK expression in the SN38-resistant subclones. (A) Quantitative real-time RT-PCR analysis of DAPK mRNA expression. (B) Western blot analysis of DAPK protein expression. Five of the seven SN38-resistant subclones (*) exhibit higher DAPK mRNA and DAPK protein expression levels than the parent cells. The cells indicated as P, 1, 2, 7, 8, 9, 10 and 12 at the bottom of the figure are parent, SN38r1, SN38r2, SN38r7, SN38r8, SN38r9, SN38r10 and SN38r12 cells, respectively.

Several lines of evidence have shown that hypermethylation of gene promoters is involved in drug-resistance (33–35). We previously reported that abnormal methylation of the DAPK gene promoter was present and the level of DAPK protein was markedly diminished in the ME180 cell line (25). Anticancer drug-sensitivities in endometrial cancer cells are modified by targeted knockdown of DAPK protein expression (30). Therefore, to examine the DAPK gene transcription and mRNA translation in the parent cells and SN38-resistant subclones, quantitative real-time RT-PCR and western blot analyses were carried out to investigate the mRNA and protein expression levels, respectively. As shown in Fig. 2, five of the seven SN38-resistant subclones expressed significantly higher levels of DAPK mRNA and DAPK protein than the parent cells. These findings indicate that translation of DAPK mRNA in the SN38-resistant subclones is not suppressed, in opposition to the effect in CDDP-resistant subclones (29).

### Methylation status of the DAPK promoter in the SN38-resistant subclones

**Figure 3:**
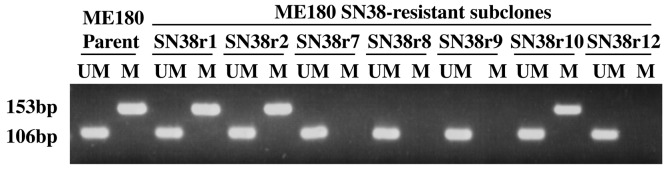
MS-DAPK-PCR analyses of the SN38-resistant subclones. Methylation-specific PCR of the DAPK gene was performed in the ME180 parent cells and seven SN38-resistant subclones. The parent cells have both the methylated and unmethylated bands. Four SN38-resistant subclones (SN38r7, SN38r8, SN38r9 and SN38r12) do not contain the methylated band. UM, unmethylated; M, methylated.

Since the parent cells and SN38-resistant subclones showed increased SN38-sensitivity upon co-treatment with SN38 and 5-aza-CdR, it is likely that the CpG islands in the DAPK gene promoter are hypermethylated in the SN38-resistant subclones. To address this possibility, we examined the methylation status of the DAPK gene promoter using DAPK-MS-PCR (Fig. 3). In four of the seven SN38-resistant subclones (SN38r7, SN38r8, SN38r9 and SN38r12), the band indicating hypermethylation of the DAPK gene promoter was not detected, while the other subclones showed the same aberrant methylation as the parent ME180 cells. Taken together, these findings indicate that there is no apparent relationship between the DAPK protein expression levels (Fig. 2) and the hypermethylation status of the DAPK gene promoter (Fig. 3) in the SN38-resistant subclones.

### Expression of DNMT genes in the SN38-resistant subclones

**Figure 4:**
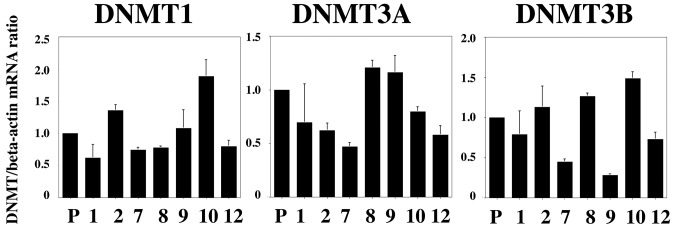
Quantitative real-time RT-PCR analyses of DNMT mRNA levels in the SN38-resistant subclones. Quantitative real-time RT-PCR analyses were carried out for three DNMT genes: DNMT1, DNMT3A and DNMT3B. The cells indicated as P, 1, 2, 7, 8, 9, 10 and 12 at the bottom of the figures are parent, SN38r1, SN38r2, SN38r7, SN38r8, SN38r9, SN38r10 and SN38r12 cells, respectively.

In our previous study, the CDDP-resistant ME180 subclones failed to maintain their restored CDDP-sensitivities after treatment with 5-aza-CdR, possibly owing to the increased DNMT3B and DNMT1 mRNA expression levels (29). To clarify whether the DNMT expression levels were also upregulated in the SN38-resistant subclones compared with the parent ME180 cells, we assessed the transcriptional expressions of the DNMT genes by quantitative real-time RT-PCR. As shown in Fig. 4, there were no relationships between the expression levels of the DNMT genes and the SN38-sensitivities in the SN38-resistant subclones, unlike the case for the CDDP-resistant subclones (29).

### Expression of DAPK protein in the parent cells and SN38-resistant subclones before and after treatment with 5-aza-CdR

As shown in Fig. 2, five of the seven SN38-resistant subclones exhibited higher DAPK protein expression levels than the parent cells. Targeted knockdown of DAPK protein expression in endometrial cancer cells was reported to enhance cellular sensitivity to 5-fluorouracil (30). To clarify whether the increased SN38-sensitivity in SN38-resistant subclones treated with 5-aza-CdR was affected by the DAPK protein expression levels after 5-aza-CdR-treatment, we examined the DAPK protein expression levels in the parent cells and SN38-resistant subclones before and after treatment with 5-aza-CdR using western blot analysis. In the parent cells, DAPK protein expression was remarkably suppressed. However, after treatment with various concentrations of 5-aza-CdR (1–10 μM) for 96 h, the parent cells showed increased DAPK protein expression in a dose-dependent manner. Similar to the case for the parent ME180 cells, two of the seven SN38-resistant subclones (SN38r1 and SN38r8) showed slightly increased DAPK protein expression after 5-aza-CdR treatment. In contrast, SN38r2 cells showed a dose-dependent reduction in DAPK protein expression after 5-aza-CdR treatment. No apparent changes in the DAPK protein expression levels were observed in the remaining four SN38-resistant subclones (SN38r7, SN38r9, SN38r10 and SN38r12).

## Discussion

Epigenetic hypermethylation of gene promoters plays an important role in tumorigenesis as an alternative mechanism for genetic changes (36). In this study, we examined the relationship between DAPK expression and SN38-resistance in ME180 cells. To evaluate the different responsiveness to SN38, we established seven monoclonal SN38-resistant subclones from ME180 cells (31), and then examined the effects of SN38 in combination with the demethylating agent 5-aza-CdR on the parent cells and SN38-resistant subclones. The SN38-sensitivities of the parent cells and the SN38-resistant subclones were all significantly enhanced by co-treatment with SN38 and 5-aza-CdR, consistent with previous findings of synergistic cytotoxicity of a combination of CDDP and 5-aza-CdR against several tumor cell lines (29,37). There are two reports showing that 5-aza-CdR enhances the SN38-cytotoxicity toward gastric carcinoma cells (38) and colorectal cancer cells (39). Moreover, we revealed that co-treatment with 5-aza-CdR overcame SN38-resistance, in addition to CDDP-resistance (29). Although co-treatment with CDDP and 5-aza-CdR enhanced the CDDP-sensitivity by less than 5-fold in the CDDP-resistant subclones (29), co-treatment with SN38 and 5-aza-CdR enhanced the SN38-sensitivity by more than 25–125-fold in the SN38-resistant subclones in the present study. This differential enhancement in the drug-sensitivities suggests that the synergistic effects of 5-aza-CdR on anticancer drug-resistant cancer cells can be much larger for SN38-resistant cells than for CDDP-resistant cells.

We also examined whether the SN38-sensitivities of the parent cells and SN38-resistant subclones were correlated with the methylation status of the DAPK gene. DAPK-MS-PCR was unable to detect a hypermethylated band in four of the seven SN38-resistant subclones, while hypermethylation of the DAPK promoter was easily detected in the remaining three SN38-resistant subclones and the parent cells. In addition, we noted that, even in the parent ME180 cells and three hypermethylation-positive SN38-resistant subclones, the methylated bands were always accompanied by unmethylated bands. However, four of the SN38-resistant subclones expressed normal unmethylated DAPK promoters. These findings are similar to those for the CDDP-resistant subclones (29), indicating that hypermethylation of the DAPK promoter in ME180 parent cells is a reversible phenomenon.

Quantitative RT-PCR analyses of DNMTs revealed no apparent relationships between the DNMT mRNA expression levels and SN38-resistance, whereas CDDP-resistance was partially modified by the DNMT expression levels in the CDDP-resistant subclones (29). The significance of the different DNMT expression levels in the two types of drug-resistant subclones remains unclear.

In contrast to ME180 parent cells, all six CDDP-resistant subclones expressed higher DAPK mRNA expressions, while five of the six CDDP-resistant subclones showed little detectable DAPK protein expression (29). In the present study, five of the seven SN38-resistant subclones exhibited higher DAPK protein expression levels than the parent cells, and the DAPK mRNA expression levels were also higher than that in the parent ME180 cells. These findings suggest that DAPK mRNA translation is strongly suppressed in the CDDP-resistant subclones, but hardly suppressed in the SN38-resistant subclones.

DAPK protein expression was induced in 5-aza-CdR-treated ME180 parent cells in a dose-dependent manner, while it was not induced by 5-aza-CdR treatment in any of the CDDP-resistant subclones (29). In the seven 5-aza-CdR-treated SN38-resistant subclones, DAPK protein expression was increased in two subclones and decreased in one subclone. Therefore, the enhanced SN38-sensitivity in the parent cells and CDDP-/SN38-resistant subclones after 5-aza-CdR treatment cannot be caused by increases or decreases in DAPK protein expression. Taking the findings of the previous report (29) and present study together, molecules demethylated by 5-aza-CdR can play important roles in the restoration of both CDDP-resistance and SN38-resistance. We are now trying to identify these molecules.

Targeted knockdown of DAPK protein expression has been reported to induce apoptosis in human differentiated endometrial adenocarcinoma cells, human endometrial carcinosarcoma cells and human uterine leiomyosarcoma cells (28). Moreover, it enhances 5-fluorouracil-sensitivity and partially enhances CDDP-sensitivity but does not enhance etoposide-sensitivity (30). These results suggest that DAPK may be a useful target molecule in molecular targeted anticancer therapies. In the present study on the human cervical squamous cancer cell line ME180, however, DAPK protein expression was strongly suppressed in the parent ME180 cells. Moreover, increases and decreases in the DAPK protein expression levels did not have direct effects on the CDDP-sensitivity and SN38-sensitivity in the ME180-derived CDDP-resistant and SN38-resistant subclones, respectively. These findings indicate that the roles of DAPK protein in cellular apoptotic signaling are cell lineage-specific and that molecular targeted anticancer therapies using suppression of DAPK expression cannot be applied to uterine cervical cancer. Our present results revealed that treatment with 5-aza-CdR could overcome the SN38-resistance in SN38-resistant subclones established from the ME180 cervical carcinoma cell line, and that neither the methylation status of the DAPK promoter nor the DAPK protein expression level was directly involved in the acquisition of SN38-resistance. The SN38-resistance may be caused by unknown changes in methylated molecules. These currently unknown molecular changes in the SN38-resistant subclones are considered to be transiently restored by demethylation treatment. Our observations suggest a possible application of treatment with a demethylating agent, such as 5-aza-CdR, to cancer therapy, which could overcome acquired SN38-resistance.

## Figures and Tables

**Figure 5:**
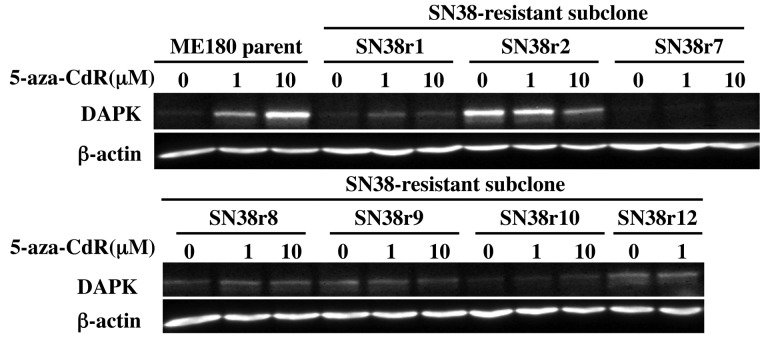
Effects of stimulation with 5-aza-CdR on DAPK protein expression in the parent cells and SN38-resistant subclones. Treatment with 5-aza-CdR clearly induces DAPK protein expression in the parent cells. Similarly, two of the seven SN38-resistant subclones (SN38r1 and SN38r8) show slightly increased DAPK protein expression after 5-aza-CdR treatment. In contrast, SN38r2 cells show a dose-dependent reduction in DAPK expression after 5-aza-CdR treatment. There are no apparent changes in the DAPK protein expression levels in the remaining four SN38-resistant subclones (SN38r7, SN38r9, SN38r10 and SN38r12).
